# Effect of diet on cognition, mental health and wellbeing among adolescents: protocol for a systematic review

**DOI:** 10.1136/bmjopen-2025-102850

**Published:** 2025-12-14

**Authors:** Joseph P Coombes, Marie Murphy, Abigail Russell, Amy Turner, Miranda Pallan

**Affiliations:** 1 University of Birmingham, Birmingham, UK; 2 NIHR School for Public Health Research, Newcastle upon Tyne, England, UK; 3 Children and Young People’s Mental Health Research Collaboration (ChYMe), University of Exeter, Exeter, UK; 4 University of Bristol, Bristol, UK

**Keywords:** Adolescents, Cognition, MENTAL HEALTH, PUBLIC HEALTH

## Abstract

**Introduction:**

A healthy diet is a crucial component for adolescents’ health and wellbeing. Current literature surrounding dietary intake and its effect on cognition, mental health and wellbeing has mainly focused on children, not adolescents. This review aims to synthesise findings from studies that explore the relationship between dietary intake and cognition, mental health and wellbeing in the adolescent population.

**Methods and analysis:**

Electronic searches will date from 1 January 2000 to 7 October 2024 and will be conducted in CENTRAL, MEDLINE/PubMed, CINAHL via EBSCOHOST, ERIC, British Education Index, Child and Adolescent Studies, Education research complete, Psychology and Behavioural Sciences Collection, Social Policy and Practice Embase, and APAPsychINFO via OvidSP. Articles will be screened using defined inclusion and exclusion criteria and assessed for eligibility by five independent reviewers. Discrepancies will be reviewed by a third reviewer. The selection process of included articles will be reported by using the Preferred Reporting Items for Systematic Reviews and Meta-Analyses flow diagram. A narrative summary will be used to report and synopsise the extracted data.

**Ethics and dissemination:**

This systematic review does not require ethical approval. The dissemination strategy for this review comprises peer-reviewed publications, public health conference presentations and providing a valuable reference for healthy-food interventions in school and community-based settings as well as identifying gaps in the current literature and informing policy and practice.

**PROSPERO registration number:**

CRD42025633083.

**STRENGTHS AND LIMITATIONS OF THIS STUDY**

- This systematic review targets literature which provides a lens into the dietary intake patterns of adolescents in relation to public health outcomes, which is currently rarely researched.
- By focusing on naturalistic settings, the included studies in this review could provide evidence for policy and practice that is more relevant to real-world contexts.
- Excluding eating behaviours as an exposure could potentially limit the insight into the dietary intake choices adolescents make.
- Due to the anticipated heterogeneity of outcome measure data, a meta-analysis is not expected to be applicable to synthesise study findings.

## Introduction

Adolescence is a vital period for biological change, brain maturation and later-life health indicators. Adolescence (ages 10–19 years) is typically a period of transition framed by physical and cognitive growth, expansion of social networks and the development of autonomous behaviour.^1 2^ This autonomous development impacts adolescents’ choice of food, the access that they have to nutrient-rich foods, and who purchases and prepares their food. A balanced nutritional intake during adolescence provides the foundation for a healthy lifestyle, and a healthy diet is fundamental to growth and cognitive development.^3 4^ A healthy diet is typically defined as a high intake of fruits, vegetables, grains and fish, which are considered to be beneficial to human health.^5^ In addition, a poor diet, or unhealthy food behaviour, is associated with several negative outcomes for children and adolescents, including an elevated risk of developing later cardiovascular disease, obesity and mental health issues.^6 7^ This relationship between food and aspects of psychological health is an emerging area of interest in research. Previous reviews have synthesised evidence on food consumption, dietary behaviour and nutritional interventions, and their relationship to cognition and mental health outcomes.^8 9^ However, there has not been a review conducted that examines these associations focusing solely on the adolescent population. Furthermore, previous reviews have not specifically examined both the acute and long-term effects of adolescents’ dietary intake on the above outcomes.

### Dietary intake and cognition

A healthy diet also has an important role in healthy cognitive processing and performance. Cognitive performance and functioning refers to processes including learning, thinking, reasoning, recall, attention and decision making.^10^ For this systematic review, these cognitive processes will be categorised into six domains of interest; Complex Attention, Learning and Memory, Language, Perceptual-Motor, Social Cognition and Executive Function. Executive functioning captures a range of processes that involve basic cognitive skills such as working memory, problem solving and attentional control. These functions play an important role in psychological health as well as academic performance.^11^

A previous systematic review published in 2016 that included 21 studies examining either food quality or macronutrient intake found consistent positive long-term effects of a healthy diet (consuming whole grains, fish, fruits and vegetables) on executive functioning in children and adolescents.^12^ One cross-sectional study included in the review reported that the consumption of sweet snacks and sugary beverages correlated in male adolescents who experienced inhibitory problems when completing decision-making tasks.^13^

Another cross-sectional study focused on the impact of breakfast consumption on adolescents’ executive function and cognitive tasks such as memory recall.^14^ It concluded that regular breakfast consumption in adolescents was associated with improved cognitive performance, and that breakfast consumption just prior to a cognitive task was associated with better task performance. Furthermore, in 2017, Burrows *et al* conducted a systematic review examining the association between dietary intake and grade point averages, school grades and reports in association with measured academic achievement in children and adolescents.^15^ From their synthesis of 33 cross-sectional studies, they concluded that consuming breakfast was reported to have positive associations with academic achievement. Conversely, negative associations were reported between fast food consumption and academic performance. Two further reviews examined the acute effects of school breakfast programmes on children and adolescents’ academic performance.^16^ The authors synthesised findings from randomised-controlled trials and concluded that breakfast consumption (relative to fasting) had short-term improvements on tasks that required attention, executive function and memory. However, firm conclusions could not be made regarding the acute effects of school-based interventions due to the limited number of studies included, which presented inconsistent findings, displaying the need to further research into the acute cognitive effects of nutritional intake in this age group.

### Dietary intake and mental health

Mental health is a multifaceted concept and is more than the absence of mental health disorders. Globally, around one in seven adolescents experiences a mental health disorder, with depression and anxiety among the leading causes of disability and illness in 10–19 year-olds.^17^ It is well recognised that adolescence is a crucial period where poor mental health symptoms can lead to adverse outcomes in areas such as educational performance, housing security and social relationships.^18^

Research has shown that consuming a low-quality diet can be associated with a myriad of mental health concerns such as depressive symptoms, emotional, conduct and hyperactive behaviours in children and adolescents.^19^,^21^ A previous systematic review of twelve studies (three cohort, prospective studies and nine cross-sectional) concluded that there was an association between a higher consumption of sweet foods and worsening mental health.^8^ From that review, a cohort study using longitudinal data from secondary schools described how the low consumption of fruit and vegetables in British adolescents correlated with poor mental health across specifically older adolescents who were from ethnic minority groups.^22^

The relationship between dietary intake and disruptive, hyperactive and aggressive behaviours (externalising disorders/behaviours) in children and adolescents has been previously explored, with studies focusing on hyperactivity. A case-control study found that, after adjustment for potential confounders, adolescents who had a poor diet quality were more likely to be diagnosed with attention deficit hyperactivity disorder (ADHD).^23^ Specifically, adolescents who did not conform to a Mediterranean diet and consumed low amounts of fruit and vegetables but had a high consumption of sugary confectionery and beverages had increased odds of a diagnosis of ADHD. There is a comparative lack of evidence and synthesis of studies that focus on internalising disorders and symptoms, such as depression and anxiety. For the purpose of this review, we are defining mental health as experiencing symptoms of anxiety and depression.

### Dietary intake and wellbeing

Wellbeing is related to but distinct from mental health and is a term that has a myriad of definitions and meanings encompassing mental wellbeing, physical wellbeing and quality of life. Generally, wellbeing is considered a positive daily state experienced by individuals and is determined by social, economic and environmental conditions.^1^ In an attempt to define adolescent wellbeing in research, an adolescent wellbeing theoretical framework has been developed, outlining five domains: (1) Good health and optimum nutrition, (2) Connectedness, positive values and contribution to society, (3) Safety and a supportive environment, (4) Learning, competence, education, skills and employability and (5) Agency and resilience.^24^ For the purpose of this review, wellbeing will be understood through two core domains, based on the established adolescent wellbeing theoretical framework: Physical Health and Psychological Health. This will encompass elements such as Psychological Wellbeing, Parent Relations and Autonomy and Social Support and Peers.

A study from New Zealand examined the associations between dietary habits and wellbeing in 8500 adolescents using the 2012 Youth national survey data. The results showed that students who consumed foods high in fat and sugar and were deemed to have unhealthy eating behaviour scored lower on the WHO Wellbeing Index outcome measure (WHO-5).^25^ Previous research on the influence of diet on the wellbeing of children and adolescents has mainly focused on eating behaviours and habits such as snacking and meal skipping, whereas there are few studies that examine dietary intake of foods and nutrients by adolescents in relation to their wellbeing.

Much of the existing research into the influence that dietary intake has on health and wellbeing has included younger children (0–9 years). Given the unique period of physical, psychological and social development that adolescents are undergoing, more evidence is needed to understand the influence dietary intake has on adolescents’ health and wellbeing, and to advance healthy eating initiatives across different contexts for policy and programme decision making.^3^

To the best of our knowledge, no systematic review has concentrated solely on the adolescent population in relation to how diet quality influences cognition, internalising mental health problems and wellbeing. Furthermore, this review aims to examine both the acute and long-term effects of adolescents’ dietary intake on cognition, mental health and wellbeing which previous reviews have not specifically done. The aim of this systematic review is to review and synthesise studies that explore the relationship between dietary intake and cognition, mental health and wellbeing in the adolescent population.

### Research questions (RQs)

The following research questions will be used to guide the review:

1. What is the association between dietary intake and cognition, mental health and wellbeing outcomes in adolescents?
2. What are the (1) Acute and (2) Long-term effects of adolescents’ dietary intake on their cognition, mental health and wellbeing?
3. What are the current research gaps and potential opportunities for future research?

## Methods

### Systematic review registration

This systematic review protocol has been registered on the PROSPERO database CRD42025633083. When first submitted for review, this systematic review was at the title and abstract screening stage (first submitted 27/03/2025). As of October 2025, data extraction has been completed and data synthesis has started, with an anticipation of completion and submission to a journal by December 2025.

### Study design

This review protocol has been written in accordance with the Preferred Reporting Items for Systematic Reviews and Meta-Analyses Protocols (PRISMA-P) statement guidelines.^26^

### Electronic searches

The search strategy was developed using the Population Exposure Context/Comparison Outcome concepts: ***Population*** (Adolescents aged 10–19 years), ***Exposure*** (Dietary Intake and Diet Quality), ***Comparison/Context*** (The comparison is drawn between varying levels or types of dietary intake (eg, high consumption vs low consumption of certain nutrients or different diet qualities/patterns) ***Outcome*** (The primary outcomes are cognition, mental health and wellbeing). After consultation with evidence synthesis experts, an initial scoping search was conducted to identify relevant literature, refine the research questions and inform the final search strategy. The following databases will be searched for articles that meet the eligibility criteria: Embase, Medline (Ovid), APA PsychInfo and Social Policy and Practice will be searched via the Ovid SP database. Alternatively, British Education Index, Child Development and Adolescent Studies, Education Research Complete, ERIC, Psychology and Behavioural Sciences Collection and CINAHL Ultimate will be searched via EBSCOHOST database. These databases contain articles that focus on psychological health from a social science perspective, research conducted in education settings and/or pupils based within those settings, and literature relating to nutritional intake and/or diet-related interventions, as well as scientific databases. The Boolean operator (AND, OR) will be used along with truncations to inform the search (search strategy provided in online supplemental appendix I). This will be followed by forward and backward citation searches of reference lists of studies that meet inclusion criteria. Articles must be published between 1 January 2000 and 7 October 2024; this will enable the identification of studies that reflect dietary intake in contemporary food environments. Hence, we focused on studies reported in the past 25 years. Included studies will be published in peer-reviewed journals and in English language only.

### Eligibility criteria

Epidemiological studies including observational studies (cross-sectional, case-control, cohort) and experimental studies (eg, randomised controlled trials, quasi-randomised trials, cluster-randomised trials) exploring the relationship between adolescent (10–19 years) dietary intake and outcomes relating to cognition, mental health and wellbeing.

#### Types of participants

Adolescent populations from all countries aged 10–19 years (as defined by the WHO).^2^

#### Types of outcome measures

**Cognition outcome measures** will include the NeuroCognitive Performance Test, d2 Test of Attention, Digit Span, Spatial Working Memory, Stroop colour-word task and any other measure that objectively measures cognitive performance (eg, memory tests, attention tests and visual perception tests) in order to capture this review’s definition of cognition and its domains.

**Mental health outcome measures** will include validated measures capturing this review’s definition of mental health (depression and/or anxiety symptoms as well as emotional problems), for example, the Child Behaviour Checklist, Strengths and Difficulties Questionnaire, Centre for Epidemiological Studies Depression Scale, Patient Health Questionnaire-9, Panic Disorder Severity Scale, Generalised Anxiety Disorder Assessment, Depression Anxiety Stress Scale, Kessler Psychological Distress Scale (K10), Youth Self Report 11–18 Questionnaire, Mood and Feelings Questionnaire, Revised Child Anxiety and Depression Scale.

**Wellbeing outcome measures** will include validated measures capturing this review’s definition of wellbeing, for example, the Warwick-Edinburgh Mental well-being Scale, Rosenberg’s Self-Esteem Scale, WHO-5, Personal well-being Index-School Children and Paediatric Quality of Life Inventory.

#### Other measures

**Dietary/nutritional intake measures** will include the Food Frequency Questionnaire and other food frequency measures, 24-hour recall, 7-day recall, Diet Diversity Score, Diet Quality Index for adolescents or any other self-reported or observed measure of dietary intake or quality (eg, researcher-recorded, parent/teacher-reported).

#### Setting

For this systematic review, we focus on naturalistic settings, which includes a variety of settings that adolescents are frequently situated in. This could include home, education or community settings.

#### Exclusion criteria

- Studies that use animals as participants.
- Studies that have a laboratory or clinical setting.
- Studies that examine associations between nutritional supplement interventions and cognition, mental health and wellbeing in the adolescent population.
- Studies that examine associations between physical health outcomes and nutritional intake and/or diet quality and/or dietary pattern.
- Studies that focus solely on eating behaviours around designated mealtimes (eg, having/skipping breakfast).
- Qualitative studies.
- Study protocols.
- Systematic and scoping reviews.
- Case series and single-case studies.
- Studies where the whole population/sample includes adolescents with a diagnosis of a physical health condition(s), mental health condition(s). Studies not published in the English language.
- Studies published before the year 2000.
- PhD theses, dissertations, conference abstracts and government reports.

### Selection of studies

Study records identified from the searches will be inputted into the CADIMA tool for title and abstract screening.^27^ Five independent reviewers (JC, AT, EA, EAG and BR) will independently screen the title and abstracts in accordance with the inclusion and exclusion criteria stated above. Some of the databases searched within this review will automatically remove duplicate articles when the extraction process takes place. However, all articles extracted into CADIMA will undergo the de-duplication process that the tool provides. In addition, the lead reviewer will review all articles where the tool highlights uncertainty about duplication and will manually de-duplicate if required. Following this process, remaining articles will be full-text screened by at least two independent reviewers, again using CADIMA. Reasons for exclusion will be recorded, and disagreements will be resolved through discussion, involving a third reviewer (MP) if required. The results of the literature search and article selection will be reported using the PRISMA flow diagram.

### Data synthesis

Two reviewers will extract information related to the following; author, title, date/year, country, aim, study design, sample size, participant characteristics, intervention characteristics (if applicable), diet measures, cognition measures, mental health measures, well-being measures, statistical presentation of results, covariates, analysis method, key findings and study strengths and weaknesses. This information will be collated using a bespoke data extraction sheet and aided by an appropriate software such as Microsoft Excel. The data extraction sheet will be piloted with three included studies and revised based on the agreement/disagreement of the two reviewers. Consequently, if a consensus is not reached, a third reviewer will be invited to provide further input.

Narrative synthesis will be used to summarise the results, due to the expected heterogeneity of studies that will meet inclusion criteria. However, if some studies are sufficiently homogeneous (eg, measuring the same aspects of diet and the same outcome measures), a meta-analysis will be conducted. The narrative synthesis will be grouped by outcomes of interest and will be summarised by measures of associations between dietary and outcome measures. If the data is sufficiently homogenous in terms of study design, exposure and outcome measures, there will also be a synthesis at the acute and long-term outcome level as well as a subgroup analysis of dietary exposures measured (eg, overall diet quality, specific macronutrient/food group intakes (fruit, vegetables, fibre, free sugar, fat etc). Once included studies have been synthesised and the first two research questions answered, the data will be interrogated to understand where there are gaps in existing knowledge and research, to answer the third research question and provide direction to those working in this field. Furthermore, systematic mapping methods will be explored if sufficient studies meet inclusion criteria for a mapping process to be useful and informative in addressing RQ3.

### Appraisal of study quality

To assess the quality of included randomised intervention studies, at least two independent reviewers will use the validated Joanna Briggs Institute (JBI) checklists. For randomised intervention studies (including cluster-randomised trials), the JBI Critical Appraisal Tool for^28^ version) will be used to determine the extent to which the study addressed the possibility of bias in its design, conduct and analysis.^28^ Quasi-randomised trials and other non-RCT intervention studies will be appraised using the specific questions outlined in the JBI Checklist for Quasi-Experimental Studies (2023 iteration).^29^ Observational studies will be assessed for methodological quality using the relevant JBI critical appraisal checklists for epidemiological research: the JBI Critical Appraisal Checklist for Cohort Studies, the JBI Critical Appraisal Checklist for Case Control Studies, and the JBI Critical Appraisal Checklist for Analytical Cross-Sectional Studies.^30^ Disagreements in the assessment of quality in the included studies will be resolved by discussions with the two reviewers and with a third reviewer if necessary. In order to compare quality across different study designs, a final score of low, moderate or high risk of bias will be allocated to each included study.

### Public involvement and engagement (PI&E)

We have conducted one-to-one conversations with educational psychologists from the University of Exeter, a food technology teacher from a UK high school and adolescents aged 13–18 years from Birmingham Youth Voice and Spark UK (a mental health organisation run by young people for young people). The aim of conducting PI&E prior to commencing the review was to co-produce this review’s research questions with teachers and school-aged adolescents to ensure that they are meaningful and relevant. These conversations have also helped to identify the outcomes of interest in this review.

### Ethics and dissemination

Ethical approval for the systematic review is not necessary as no human participants will be directly involved. The findings of this review will inform the first work package of a PhD study which aims to examine the acute effects that school food has on pupils’ short-term outcomes such as attention, concentration, mental health, wellbeing and behaviour. The findings of this review will be presented at public health conferences and published as one peer-reviewed journal article.

Extracted data will not be made open access; however, the lead author can be contacted for access to this dataset.

## Supplementary material

10.1136/bmjopen-2025-102850 online supplemental file 1

## References

[1] Ziegler AM, Kasprzak CM, Mansouri TH (2021). An Ecological Perspective of Food Choice and Eating Autonomy Among Adolescents. Front Psychol.

[2] World Health Organisation (2024). Adolescent health. https://www.who.int/health-topics/adolescent-health#tab=tab_1.

[3] Neufeld LM, Andrade EB, Ballonoff Suleiman A (2022). Food choice in transition: adolescent autonomy, agency, and the food environment. The Lancet.

[4] Aurino E (2017). Do boys eat better than girls in India? Longitudinal evidence on dietary diversity and food consumption disparities among children and adolescents. Econ Hum Biol.

[5] Brouwer ID, van Liere MJ, de Brauw A (2021). Reverse thinking: taking a healthy diet perspective towards food systems transformations. Food Sec.

[6] Ruiz LD, Zuelch ML, Dimitratos SM (2020). Adolescent Obesity: Diet Quality, Psychosocial Health, and Cardiometabolic Risk Factors. Nutrients.

[7] Çağiran Yilmaz F, Çağiran D, Özçelik AÖ (2019). Adolescent Obesity and Its Association with Diet Quality and Cardiovascular Risk Factors. Ecol Food Nutr.

[8] O’Neil A, Quirk SE, Housden S (2014). Relationship Between Diet and Mental Health in Children and Adolescents: A Systematic Review. Am J Public Health.

[9] Roberts M, Tolar-Peterson T, Reynolds A (2022). The Effects of Nutritional Interventions on the Cognitive Development of Preschool-Age Children: A Systematic Review. Nutrients.

[10] Taphoorn MJB, Klein M (2012). Evaluation of cognitive functions and quality of life. Handb Clin Neurol.

[11] Henriksson P, Cuenca-García M, Labayen I (2017). Diet quality and attention capacity in European adolescents: the Healthy Lifestyle in Europe by Nutrition in Adolescence (HELENA) study. Br J Nutr.

[12] Cohen JFW, Gorski MT, Gruber SA (2016). The effect of healthy dietary consumption on executive cognitive functioning in children and adolescents: a systematic review. Br J Nutr.

[13] Ames SL, Kisbu-Sakarya Y, Reynolds KD (2014). Inhibitory control effects in adolescent binge eating and consumption of sugar-sweetened beverages and snacks. Appetite.

[14] Peña-Jorquera H, Campos-Núñez V, Sadarangani KP (2021). Breakfast: A Crucial Meal for Adolescents’ Cognitive Performance According to Their Nutritional Status. The Cogni-Action Project. Nutrients.

[15] Burrows T, Goldman S, Pursey K (2017). Is there an association between dietary intake and academic achievement: a systematic review. J Hum Nutr Diet.

[16] Adolphus K, Lawton CL, Champ CL (2016). The Effects of Breakfast and Breakfast Composition on Cognition in Children and Adolescents: A Systematic Review. *Adv Nutr*.

[17] World Health Organization (WHO) (2021). Mental health of adolescents. https://www.who.int/news-room/fact-sheets/detail/adolescent-mental-health.

[18] Alegría M, NeMoyer A, Falgàs Bagué I (2018). Social Determinants of Mental Health: Where We Are and Where We Need to Go. Curr Psychiatry Rep.

[19] Sinclair R, Millar L, Allender S (2016). The Cross-Sectional Association between Diet Quality and Depressive Symptomology amongst Fijian Adolescents. PLoS ONE.

[20] Robinson M, Kendall GE, Jacoby P (2011). Lifestyle and demographic correlates of poor mental health in early adolescence. J Paediatr Child Health.

[21] Kohlboeck G, Sausenthaler S, Standl M (2012). Food intake, diet quality and behavioral problems in children: results from the GINI-plus/LISA-plus studies. Ann Nutr Metab.

[22] Huang P, O’Keeffe M, Elia C (2019). Fruit and vegetable consumption and mental health across adolescence: evidence from a diverse urban British cohort study. Int J Behav Nutr Phys Act.

[23] Ríos-Hernández A, Alda JA, Farran-Codina A (2017). The Mediterranean Diet and ADHD in Children and Adolescents. Pediatrics.

[24] Ross DA, Hinton R, Melles-Brewer M (2020). Adolescent Well-Being: A Definition and Conceptual Framework. J Adolesc Health.

[25] Puloka ‘Ilaisaane, Utter J, Denny S (2017). Dietary behaviours and the mental well‐being of New Zealand adolescents. J Paediatrics Child Health.

[26] Moher D, Shamseer L, Clarke M (2016). Preferred reporting items for systematic review and meta-analysis protocols (PRISMA-P) 2015 statement. Revista Espanola de Nutricion Humana y Dietetica.

[27] Kohl C, McIntosh EJ, Unger S (2018). Online tools supporting the conduct and reporting of systematic reviews and systematic maps: a case study on CADIMA and review of existing tools. Environ Evid.

[28] Barker TH, Stone JC, Sears K (2023). The revised JBI critical appraisal tool for the assessment of risk of bias for randomized controlled trials. JBI Evid Synth.

[29] Barker TH, Habibi N, Aromataris E (2024). The revised JBI critical appraisal tool for the assessment of risk of bias for quasi-experimental studies. JBI Evid Synth.

[30] Moola S, Munn Z, Tufanaru C (2020). JBI manual for evidence synthesis.

