# Supplementary material for: Effect of diet on cognition, mental health and wellbeing among adolescents: protocol for a systematic review
Source: BMJ Open. 2025 Dec 14;15(12):e102850. doi: 10.1136/bmjopen-2025-102850 (PMC12706249; doi:10.1136/bmjopen-2025-102850)
Supplement: online supplemental file 1 [file bmjopen-15-12-s001.docx]

Search strategies

(Databases – Embase, Medline (OViD), APA PsychInfo, Social Policy & Practice)

*Cognition*

1. (Adolescen* or teen* or pupil* or "young people" or youth).ti,ab.

2. limit 1 to english language

3. limit 2 to human

4. limit 3 to humans

5. limit 4 to yr="2000 -Current"

6. (Cognition or "executive function" or memory or attention) ti,ab.

7. limit 6 to english language

8. limit 7 to human

9. limit 8 to humans

10. limit 9 to yr="2000 -Current"

11. (Diet or "dietary intake" or "nutritional Intake" or "food consumption" or "food intake").ti,ab.

12. limit 11 to english language

13. limit 12 to human

14. limit 13 to humans

15. limit 14 to yr="2000 -Current"

16. 5 and 10 and 15

17. remove duplicates from 16

*Mental Health*

1. (Adolescen* or teen* or pupil* or "young people" or youth).ti,ab.

2. limit 1 to english language

3. limit 2 to human

4. limit 3 to humans

5. limit 4 to yr="2000 -Current"

6. (Mental* or "mental health" or "mental process*" or anxi* or "mental stress" or depression or "depressive symptom*").ti,ab.

7. limit 6 to english language

8. limit 7 to human

9. limit 8 to humans

10. limit 9 to yr="2000 -Current"

11. (Diet or "dietary intake" or "nutritional Intake" or "food consumption" or "food intake").ti,ab.

12. limit 11 to english language

13. limit 12 to human

14. limit 13 to humans

15. limit 14 to yr="2000 -Current"

16. 5 and 10 and 15

17. remove duplicates from 16

*Wellbeing*

1. (Adolescen* or teen* or pupil* or "young people" or youth).ti,ab.

2. limit 1 to english language

3. limit 2 to human

4. limit 3 to humans

5. limit 4 to yr="2000 -Current"

6. (Well-being or "Mental Well-being" or "Social Well-being" or "Emotional Well-being").ti,ab.

7. limit 6 to english language

8. limit 7 to human

9. limit 8 to humans

10. limit 9 to yr="2000 -Current"

11. (Diet or "dietary intake" or "nutritional Intake" or "food consumption" or "food intake").ti,ab.

12. limit 11 to english language

13. limit 12 to human

14. limit 13 to humans

15. limit 14 to yr="2000 -Current"

16. 5 and 10 and 15

17. remove duplicates from 16

EBSCOhost databases (British Education Index, Child development & adolescent studies, Education Research Complete, ERIC, psychology & behavioural sciences collection, and CINAHL Ultimate).

*Cognition*

1. TX (Adolescen* or teen* or pupil* or "young people" or youth)

2. limit 1 to english language

3. limit 2 to human

4. limit 3 to humans

5. limit 4 to yr="2000 -Current"

6. TX (Cognition or "executive function" or memory or attention)

7. limit 6 to english language

8. limit 7 to human

9. limit 8 to humans

10. limit 9 to yr="2000 -Current"

11. TX (Diet or "dietary intake" or "nutritional Intake" or "food consumption" or "food intake")

12. limit 11 to english language

13. limit 12 to human

14. limit 13 to humans

15. limit 14 to yr="2000 -Current"

16. 5 and 10 and 15

17. Limit 16 to Source Types = Academic Journals

*Mental Health*

1. TX (Adolescen* or teen* or pupil* or "young people" or youth)

2. limit 1 to english language

3. limit 2 to human

4. limit 3 to humans

5. limit 4 to yr="2000 -Current"

6. TX (Mental* or "mental health" or "mental process*" or anxi* or "mental stress" or depression or "depressive symptom*")

7. limit 6 to english language

8. limit 7 to human

9. limit 8 to humans

10. limit 9 to yr="2000 -Current"

11. TX (Diet or "dietary intake" or "nutritional Intake" or "food consumption" or "food intake")

12. limit 11 to english language

13. limit 12 to human

14. limit 13 to humans

15. limit 14 to yr="2000 -Current"

16. 5 and 10 and 15

17. Limit 16 to Source Types = Academic Journals

*Wellbeing*

1. TX (Adolescen* or teen* or pupil* or "young people" or youth)

2. limit 1 to english language

3. limit 2 to human

4. limit 3 to humans

5. limit 4 to yr="2000 -Current"

6. TX (Well-being or "Mental Well-being" or "Social Well-being" or "Emotional Well-being")

7. limit 6 to english language

8. limit 7 to human

9. limit 8 to humans

10. limit 9 to yr="2000 -Current"

11. TX (Diet or "dietary intake" or "nutritional Intake" or "food consumption" or "food intake")

12. limit 11 to english language

13. limit 12 to human

14. limit 13 to humans

15. limit 14 to yr="2000 -Current"

16. 5 and 10 and 15

17. Limit 16 to Source Types = Academic Journals
