# Supplementary material for: Effect of diet on cognition, mental health and wellbeing among adolescents: protocol for a systematic review
Source: BMJ Open. 2025 Dec 14;15(12):e102850. doi: 10.1136/bmjopen-2025-102850 (PMC12706249; doi:10.1136/bmjopen-2025-102850)
Supplement: Supplementary file 2 [file bmjopen-2025-102850.reviewer_comments.pdf]

PEER REVIEW HISTORY

BMJ Open publishes all reviews undertaken for accepted manuscripts. Reviewers are asked to complete a checklist review form and are provided with free text boxes to elaborate on their assessment. These free text comments are reproduced below.

ARTICLE DETAILS

Title (Provisional)

Effect of diet on cognition, mental health and wellbeing among adolescents: protocol for a systematic review

Authors

Coombes, Joseph; Murphy, Marie; Russell, Abigail; Turner, Amy; Pallan, Miranda

---

VERSION 1 - REVIEW

**Reviewer**	**1**
**Name**	**Okekunle, Akinkunmi Paul**
**Affiliation**	**University of Ibadan**
**Date**	**25-Jun-2025**
**COI**	**None**

Coombes et al. developed a systematic review protocol on the question of "How does the dietary intake of adolescents affect their cognition, mental health and wellbeing? A systematic review protocol". This is a worthy cause, and there is scientific merit in making a case for the protocol. However, several aspect of the methodology needs to be clarified, especially concerning the criteria for inclusion and exclusion. In the same vein, there is currently a lack of clarity on a solid and robust method(s) for evidence synthesis to inform the rigour and scientific validity of the findings. Additionally, the current protocol does not itemise the expected findings in the context of potential challenges likely to be encountered and how these would be addressed during the review. A point-by-point outline of comments along the protocol presentation is presented below;

Abstract

Page 1

1. "Electronic searches will date from 1/1/2000 to 7/10/2024" What is the basis for the limited search period?.

2. "The selection process of included articles will be recorded by using the Preferred Reporting Items for Systematic Reviews and Meta-Analyses (PRISMA) flow diagram." It is

advisable to revise this statement, as the PRISMA is not a recording tool but a reporting tool for guiding systematic reviews

3. "A narrative summary will be …" Beyond narrative synthesis, authors should consider other forms of evidence synthesis for narrative review: JBA, Campbell EGM guidelines, etc.

4. Was the protocol for the review registered in a systematic protocol registry? If not, the author can consider this.

Introduction

5. The introduction was quite exhaustive and detailed. However, the author omitted contextualizing the currently proposed review in the light of previous efforts. What would this proposed review be adding? How is it different from previous efforts? What would be different that has not been done before? Would this confirmatory evaluation of previous findings?

Methods

6. PICO principles are critical for systematic reviews. This is not clarified in the proposed methods, as it forms the primary framework to guide the review.

7. Has there been any primary or initial literature search to ensure the availability of articles for the review?

8. Page 5, "1/1/2000 to 07 /10/2024" why is the search period limited?

Page 6:

9. "evaluation studies" is better represented as experimental studies or clinical trials.

10. The eligibility criteria are not detailed, and some infusing some rigour would be necessary. For example, the authors planned to consider "Adolescents aged 10 -19 years," but did not consider the setting of the study design, recruitment, and other confounding factors. This age criterion is without specificity. Would this also include adolescents under juvenile restrictive conditions? OR those under a regimented or controlled environment. These factors, along with others, play a role in determining the type of studies to include to avoid qualitative heterogeneity and confounding.

11. Similarly, there is no clarity about the "Cognition outcome measures". The criteria are too vague. Studies to be included are likely to encompass diverse measures of cognition, and how would these be harmonized and interpreted in the course of the review?

12. The same point applies for "Mental health outcome measures", "Wellbeing outcome measures", etc.

Page 7:

13. "Exclusion criteria" focused primarily on the expected exclusion, especially evidence from some non-peer-reviewed literature. However, critical criteria, such as population

differences and other factors related to participant type, recruitment, etc., were not accounted for.

14. "Selection of studies." It is also crucial for the author to screen the references of included articles for potential additional articles to be included in the review.

15. "The results of the literature search and article selection will be presented using the Preferred Reporting Items for Systematic Reviews and Meta-Analyses (PRISMA) flow diagram." This statement is inaccurate, as PRISMA guidelines are reporting tools. Please consider revising it.

16. Page 7, lines 34 – 38: In terms of the timing of medication use, how were respondents with short periods of medication use (< 5 years) handled? It is imperative because medication use is vital for hypertension prognosis.

17. Page 8, "Narrative synthesis will be used to summarise the results, due to the expected heterogeneity of studies that will meet inclusion criteria." The authors may want to consider additional scientific approaches for evidence synthesis to ensure the robustness of their findings. Such include: JBA, Campbell EGM guidelines, etc.

18. A section on expected results, including potential confounding issues likely to arise during the review, and how the authors plan to address them, would be necessary.

19. In addition, what are the likely limitations in the conduct and reporting of the review?

20. “Appendix I: Search strategy” The search strategy is very scanty and vague. The authors would benefit from bringing on board an evidence synthesis specialist, information specialist, or librarian for the review.

**Reviewer**	**2**
**Name**	**Salama, Amany**
**Affiliation**	**Pharos University, Nutrition and food safty**
**Date**	**12-Jul-2025**
**COI**	**None**

The following areas require more clarity to ensure methodological rigor and reproducibility:

The protocol must include a draft search strategy for at least one key database (e.g., MEDLINE via OvidSP or PubMed) showing the exact terms and structure. State planned filters explicitly (e.g., LIMIT 2000-01-01:2024-10-07, English language).

Study Selection Process:

It states two reviewers will screen, but lacks detail on how disagreements will be resolved.

Improvement Needed: Explicitly state the conflict resolution process (e.g., "Disagreements at title/abstract or full-text screening stage will be resolved through discussion or, if unresolved, adjudication by a third reviewer (XX)").

Outcome Definition and Measurement:

Outcomes ("cognition, mental health and wellbeing") are stated but not defined or operationalized. What specific cognitive domains (e.g., memory, executive function)? What aspects of mental health (e.g., depression, anxiety symptoms, diagnosed disorders)? What measures of well-being (e.g., life satisfaction, quality of life)? Which validated tools/questionnaires are acceptable?

Improvement Needed: Provide clear, detailed definitions for each outcome category. List specific constructs and acceptable measurement tools/scales. Specify primary and secondary outcomes if applicable.

Data Synthesis Methodology:

It states that a "narrative summary" will be used, but provides no detail on how the narrative synthesis will be conducted. Crucially, it doesn't address the possibility or plan for meta-analysis.

Improvement Needed: Detail the narrative synthesis approach (e.g., grouping studies by exposure/outcome, describing patterns). Explicitly state the criteria for deciding if meta-analysis is appropriate (e.g., sufficient homogeneity in PICO, outcome measures, and study quality). If meta-analysis is possible, describe planned methods (e.g., effect measures like OR, RR, MD; fixed/random effects models; heterogeneity assessment like I^2 statistic; software like RevMan or R).

Risk of Bias (Quality) Assessment:

The protocol does not mention assessing the risk of bias or quality of included studies at all. This is a critical omission for a systematic review.

Improvement Needed: Specify the tool(s) that will be used to assess risk of bias (e.g., Cochrane RoB 2 for RCTs, ROBINS-I for non-randomized studies, Newcastle-Ottawa Scale for observational studies). Describe the process (e.g., independent assessment by two reviewers, resolution of disagreements). Describe how this assessment will influence the synthesis. (e.g., sensitivity analysis excluding high RoB studies).

Inclusion/Exclusion Criteria Detail:

The abstract mentions "defined inclusion and exclusion criteria," but these are not detailed in the provided content. Specifics regarding study designs, population (e.g., age range defining "adolescent"), exposure (e.g., specific dietary patterns, nutrients), comparators, and outcomes are essential.

Improvement Needed: Explicitly list the inclusion/exclusion criteria within the protocol body (e.g., "Included: RCTs, cohort, cross-sectional studies; Participants aged 10-19 years; Studies

measuring dietary intake (e.g., FFQ, 24hr recall) and at least one cognitive/mental health/wellbeing outcome; Published in English from 2000-present. Excluded: Studies focusing solely on clinical populations or supplementation...").

The protocol aims to inform "healthy-food interventions in school and community-based settings" and "policy and practice." Unclear methods directly jeopardize this goal. The broad outcome terms ("cognition, mental health, wellbeing") without definitions make inconsistency inevitable.

The lack of detail on synthesis and quality assessment means the review's conclusions could be misleading or unusable for its intended audience (public health practitioners, policymakers, schools).

Addressing these areas in the protocol itself is essential to ensure the systematic review produces reliable evidence to improve adolescent health.

VERSION 1 - AUTHOR RESPONSE

Responses to Reviewer 1 (Dr. Akinkunmi Paul Okekunle)

Abstract

**Comment Number**	**Reviewer Query**	**Response**	**Citation/Action Taken**
1 (abstract) & 8 (methods)	What is the basis for the limited search period (1/1/2000 to 7/10/2024)?.	The protocol specifies that this time limit was chosen to **"enable the identification of recent high-quality studies that will be most beneficial for practice and policy decision makers"** stated under the heading 'Electronic searches' Page 8.	The sentence **"this will enable the identification of recent high-quality studies that will be most beneficial for practice and policy decision makers."** on Page 8 under the heading 'Electronic searches' will remain.
2 (abstract) & 15 (methods)	"The selection process of included articles will be recorded by using the Preferred Reporting Items for Systematic Reviews and Meta-Analyses (PRISMA) flow diagram." It is advisable to revise this statement, as the PRISMA is not a recording tool but a	We agree. The relevant sentences in the protocol have been revised to clarify that the results of the literature search will be presented using the PRISMA flow diagram, while the CADIMA tool will be used for the recording of the screening process.	Abstract amended on Page 1 and Methods section (under the heading 'Study design) on Page 5 amended to reflect the reviewers comments.

	reporting tool for guiding systematic reviews		
3 (abstract) & 17 (methods Page 8)	Beyond narrative synthesis, authors should consider other forms of evidence synthesis (JBA, Campbell EGM guidelines, etc.).	We appreciate this suggestion however, the protocol already specifies “that while narrative synthesis is the primary approach due to expected heterogeneity, meta-analysis will be conducted if studies are sufficiently homogeneous.”	The protocol details plans for narrative synthesis grouped by outcome and exposure and potential meta-analysis if the data is sufficiently homogeneous. Page 8 under the heading data synthesis
4 (abstract)	Was the protocol for the review registered?	Yes, the protocol has been registered. The registration details were provided in the Methods section (Page 5 – under the heading ‘Systematic review registration) **"This systematic review protocol has been registered on the PROSPERO database CRD42025633083"**.	The PROSPERO registration details have been added to the abstract. (Page 1).

Introduction

(5)	The introduction was quite exhaustive and detailed. However, the author omitted contextualizing the currently proposed review in the light of previous efforts. What would this proposed review be adding? How is it different from previous efforts? What would be different that has not been done before? Would this confirmatory evaluation of previous findings?	The introduction clarifies that previous reviews have mainly focused on younger children (0-9 years) or have not concentrated solely on the adolescent population (ages 10-19 years). This review is unique because it specifically addresses the influence of diet quality on cognition, mental health, and wellbeing in adolescents, and specifically aims to explore both the acute and long-term effects.	As the introduction already states the novel element of this review, we have added a sentence in order define in a clearer manner what this review will add.

Methods

Comment Number	Reviewer Query	Response	Citation/Action Taken
6	PICO principles are critical for systematic reviews. This is not clarified.	We agree. Although the elements (Population, Exposure/Intervention, and Outcomes) are defined in the eligibility criteria, we will add an explicit PICO/PECO framework statement at the beginning of the Methods section to guide the review structure.	PECO concepts added on Page 6 at the start of the methods section under the heading 'Electronic searches'. Further PECO elements are fully described in the text in the methods section.
7	Has there been any primary or initial literature search to ensure the availability of articles?	Yes, initial scoping and consultation with search experts were conducted to develop the strategy and ensure the viability of the review question.	A sentence has been added to the section 'Electronic searches' detailing the scoping searches that were undertaken and the date they were conducted.
9	"evaluation studies" is better represented as experimental studies or clinical trials.	We accept this suggested revision for improved clarity. The term **"evaluation studies"** will be replaced with **"experimental studies (e.g., randomised controlled trials, quasi-randomised trials, cluster-randomised trials)"**.	The protocol now states the term experimental studies instead of evaluation. Page 6 – Methods section under the heading 'Eligibility criteria'.
10	The eligibility criteria are not detailed, and some infusing some rigour would be necessary. For example, the authors planned to consider "Adolescents aged 10 - 19 years," but did not consider the setting of the study design, recruitment, and other confounding factors. This age criterion is without specificity. Would this also include adolescents under juvenile restrictive	We have detailed the eligibility criteria by defining the population as adolescents aged 10-19 years (WHO definition). We focus on naturalistic settings (home, education, community) and exclude laboratory or clinical settings, or studies focusing solely on clinical populations or physical/mental health conditions. This explicitly excludes regimented or restrictive clinical environments. We also state that confounding factors will be addressed by extracting relevant covariates during data	Eligibility criteria, Types of participant, setting and exclusion criteria headings and information on pages 6 & 7.

	conditions? OR those under a regimented or controlled environment. These factors, along with others, play a role in determining the type of studies to include to avoid qualitative heterogeneity and confounding.	extraction and addressing their impact in the narrative synthesis or sensitivity analysis.	
11 & 12	Similarly, there is no clarity about the "Cognition outcome measures". The criteria are too vague. Studies to be included are likely to encompass diverse measures of cognition, and how would these be harmonized and interpreted in the course of the review? 12. The same point applies for "Mental health outcome measures", "Wellbeing outcome measures", etc.	The protocol explicitly lists numerous validated tools that will be accepted for measuring these outcomes, such as the NeuroCognitive Performance Test (NCPT) for cognition, the Child Behaviour Checklist (CBCL) for mental health. In terms of wellbeing the definition has been updated in the introduction of this protocol. This has also been reflected in the wellbeing measures section which now encompasses the domains we are interested in, in specific outcome measures.<br><br>We will provide clearer narrative definitions for the specific constructs captured by these measures (e.g., defining cognition domains as memory, executive function, attention).	We have provided a clearer narrative definition for wellbeing in the introduction of this protocol as well as in the outcome section. Also, we have done this for the other two outcomes for the specific constructs captured by these measures (e.g., defining cognition domains as memory, executive function, attention) to harmonise interpretation, as noted in the data synthesis plan.
13	"Exclusion criteria" focused primarily on the expected exclusion, especially evidence from some non-peer-reviewed literature. However, critical criteria, such as population differences and other factors related to participant type, recruitment, etc.,	The exclusion criteria already address population differences by explicitly excluding studies focusing solely on animals, laboratory/clinical settings, or populations with a diagnosis of a physical or mental health condition. Any remaining population differences in included studies will be managed through subgroup analysis if data allows, or	We have added to the eligibility criteria under the heading 'Types of participants' on Page 6 that adolescent populations from all countries will be eligible for inclusion in this review.

	were not accounted for.	through detailed description in the narrative synthesis.	
14	"Selection of studies." It is also crucial for the author to screen the references of included articles for potential additional articles to be included in the review.	This process is already included in the protocol: **"This will be followed by forward and backward citation searches of reference lists of studies that meet inclusion criteria"**.	See Electronic searches section in Methods section – Page 5.
15	"The results of the literature search and article selection will be presented using the Preferred Reporting Items for Systematic Reviews and Meta-Analyses (PRISMA) flow diagram." This statement is inaccurate, as PRISMA guidelines are reporting tools. Please consider revising it.	We agree. The relevant sentences in the protocol have been revised to clarify that the results of the literature search will be presented using the PRISMA flow diagram, while the CADIMA tool will be used for the recording of the screening process.	Methods section (under the heading 'Study design) on Page 5 amended to reflect the reviewers' comments.
16	Page 7, lines 34 – 38: In terms of the timing of medication use, how were respondents with short periods of medication use (< 5 years) handled? It is imperative because medication use is vital for hypertension prognosis.	This review is not looking at medication or hypertension.	N/A
17	Page 8, "Narrative synthesis will be used to summarise the results, due to the expected heterogeneity of studies that will meet inclusion criteria." The authors may want to consider additional scientific approaches for evidence synthesis to ensure the robustness of their	We appreciate this suggestion. The protocol already specifies "that while narrative synthesis is the primary approach due to expected heterogeneity, meta-analysis will be conducted if studies are sufficiently homogeneous."	The protocol details plans for narrative synthesis grouped by outcome and exposure and potential meta-analysis if the data is sufficiently homogeneous. Page 8 under the heading data synthesis.

	findings. Such include: JBA, Campbell EGM guidelines, etc.		
18	A section on expected results, including potential confounding issues likely to arise during the review, and how the authors plan to address them, would be necessary.	Confounding factors will be fully detailed in the review write-up. Expected results in a protocol is not usual practice. This protocol outlines the narrative synthesis plan **"will be grouped by outcomes of interest: initially in broad categories of cognition, mental health, and wellbeing, and potentially in finer-grained detail if sufficient studies are included. If the data allow, there will also be a synthesis at the acute and long-term-outcome level as well as a subgroup analysis of dietary exposures measured (e.g., overall diet quality, specific macronutrient/food group intakes (fruit, vegetables, fibre, free sugar, fat etc.)."**	The full completed systematic review write-up will detail all confounding factors.
19	In addition, what are the likely limitations in the conduct and reporting of the review?	As per normal practice, full details of limitations in this review will be provided in the review write-up. The section titled 'STRENGTHS AND LIMITATIONS OF THE STUDY' on Page 2 details the expected limitations of this review.	Limitations section can be found on Page 2 - titled 'STRENGTHS AND LIMITATIONS OF THE STUDY'.
20	Search strategy is very scanty and vague. Consult specialist.	We acknowledge the current search appendix is brief. The attached appendix highlights detailed search strategies for each database searched. confirmed that search and review experts, including the Evidence Synthesis team, were consulted during the development of the strategy. The search strategies will be provided as a supplementary file, addressing this concern and Editorial Comment 6.	Appendix I now reflects detailed search strategies of each database.

Responses to Reviewer 2 (Dr. Amany Salama)

Comment Number	Reviewer Query	Response	Citation/Action Taken
1. Draft Search Strategy	The protocol must include a draft search strategy for at least one key database (e.g., MEDLINE via OvidSP or PubMed) showing the exact terms and structure. State planned filters explicitly (e.g., LIMIT 2000-01-01:2024-10-07, English language).	Parts of the search strategy are detailed in the abstract (Page 1) and a more detailed account in the Electronic searches heading in the Methods section where filters and date limits are defined. (Page 5). The full search strategy will be provided in the appendix (attached file).	Appendix I now reflects detailed search strategies of each database.
2. Study Selection Process	It states two reviewers will screen, but lacks detail on how disagreements will be resolved.<br><br>Improvement Needed: Explicitly state the conflict resolution process (e.g., "Disagreements at title/abstract or full-text screening stage will be resolved through discussion or, if unresolved, adjudication by a third reviewer (XX)").	The protocol details the conflict resolution process: **"disagreements will be resolved through discussion, involving a third reviewer if required"**. We will clarify this further by explicitly naming the third reviewer (by initials).	Third reviewer initials added in the 'Selection of Studies' heading within the Methods section on Page 7.
3. Outcome Definition and Measurem ent	Outcomes ("cognition, mental health and wellbeing") are stated but not defined or operationalized. What specific cognitive domains (e.g., memory, executive function)? What aspects of mental health (e.g., depression, anxiety symptoms, diagnosed disorders)? What measures of well-being (e.g., life satisfaction, quality of life)? Which validated tools/questionnaires are acceptable?<br>Improvement Needed:	The protocol lists validated tools for each outcome and defines each outcome within the introduction text. We will add more detail around the definitions specifying the each outcome in the introduction.<br><br>This has also been reflected in the wellbeing measures section which now encompasses the domains we are interested in, in specific outcome measures.<br>We will provide clearer narrative definitions for the specific constructs captured by these measures (e.g., defining	Specific tools are listed for Cognition, Mental Health, and Wellbeing. We have provided a clearer narrative definition for cognition, mental health and wellbeing in the introduction of this protocol as well as in the outcome section. Also we have done this for the other two outcomes for the specific constructs captured by these measures (e.g., defining cognition domains as memory, executive function, attention) to

Commented [JC1]: Or should the real specificity around the outcomes go in the outcome measure section?

	Provide clear, detailed definitions for each outcome category. List specific constructs and acceptable measurement tools/scales. Specify primary and secondary outcomes if applicable.	cognition domains as memory, executive function, attention)	harmonise interpretation, as noted in the data synthesis plan.
4. Data Synthesis Methodology	It states that a "narrative summary" will be used, but provides no detail on how the narrative synthesis will be conducted. Crucially, it doesn't address the possibility or plan for meta-analysis.<br>Improvement Needed: Detail the narrative synthesis approach (e.g., grouping studies by exposure/outcome, describing patterns). Explicitly state the criteria for deciding if meta-analysis is appropriate (e.g., sufficient homogeneity in PICO, outcome measures, and study quality). If meta-analysis is possible, describe planned methods (e.g., effect measures like OR, RR, MD; fixed/random effects models; heterogeneity assessment like I^2 statistic; software like RevMan or R).	The protocol explicitly states on page 8 under the heading 'Data Synthesis' how the narrative synthesis will be detailed: grouped by outcomes (cognition, mental health, wellbeing), and further synthesised by acute/long-term outcome level and dietary exposure subgroups (e.g., diet quality, specific macronutrients/food groups). The protocol does address meta-analysis, stating it will be conducted "if some studies are sufficiently homogeneous" (e.g., measuring the same aspects of diet and outcomes).	The protocol already states the reviewers' queries in-text as stated in the response column.<br>We have amended/added the sentence 'sufficiently homogenous in terms of study design, exposure and outcome measures' to the section 'Data Synthesis' on Page 8.
5. Risk of Bias (Quality) Assessment	The protocol does not mention assessing the risk of bias or quality of included studies at all. This is a critical omission for a systematic review.<br>Improvement Needed: Specify the tool(s) that will be used to assess risk of	The protocol explicitly details in the 'Appraisal of study quality' section (pages 8-9). We specified the tools based on study design: Revised Cochrane risk-of-bias tool for randomised trials (RoB 2), ROBINS-I for non-RCT interventions, and ROBINS-E for observational studies. The	A risk of bias section was already present in the protocol (pages 8-9). We have updated that section to reflect the risk of bias tools we will now be using – Joanna Briggs Institute checklists for all of our

	bias (e.g., Cochrane RoB 2 for RCTs, ROBINS-I for non-randomized studies, Newcastle-Ottawa Scale for observational studies). Describe the process (e.g., independent assessment by two reviewers, resolution of disagreements). Describe how this assessment will influence the synthesis. (e.g., sensitivity analysis excluding high RoB studies).	process involves two independent reviewers resolving disagreement through discussion/adjudication. We will explicitly state how this assessment will influence the synthesis, for instance, by conducting a sensitivity analysis excluding studies deemed high risk of bias.<br><br>*To note – we have update the Risk of bias section as the review will now be using the Joanna Briggs Institute checklist to assess risk of bias.	included study methodologies.
6. Inclusion/Exclusion Criteria Detail	The abstract mentions "defined inclusion and exclusion criteria," but these are not detailed in the provided content. Specifics regarding study designs, population (e.g., age range defining "adolescent"), exposure (e.g., specific dietary patterns, nutrients), comparators, and outcomes are essential. Improvement Needed: Explicitly list the inclusion/exclusion criteria within the protocol body (e.g., "Included: RCTs, cohort, cross-sectional studies; Participants aged 10-19 years; Studies measuring dietary intake (e.g., FFQ, 24hr recall) and at least one cognitive/mental health/wellbeing outcome; Published in English from 2000-present. Excluded: Studies focusing solely on clinical populations or supplementation...").	The protocol clearly states an eligibility criteria (page 6) and an exclusion criteria (page 7). Specifics regarding "study designs, population (e.g., age range defining "adolescent"), exposure (e.g., specific dietary patterns, nutrients), comparators, and outcomes" as the review states was included in the submitted manuscript with section headings found on pages 6-7.	The detail the reviewer requires was already in the protocol manuscript and can be found on pages 6-7.

VERSION 2 - REVIEW

**Reviewer**	**1**
**Name**	**Okekunle, Akinkunmi Paul**
**Affiliation**	**University of Ibadan**
**Date**	**05-Nov-2025**
**COI**	

Thank you for investing your time and effort in considering the comments and suggestions to revise the manuscript. Indeed, there are significant improvements. However, I have the following points for the authors to consider:

1. In reply to comment 1, the author states that “The protocol specifies that this time limit was chosen to "enable the identification of recent high-quality studies that will be most beneficial for practice and policy decision makers" stated under the heading ‘Electronic searches’ Page 8.” However, it is highly speculative and subjective to conclude that only “recent high-quality studies that will be most beneficial for practice and policy”. I suggest that the authors critically review this and revise the protocol accordingly.

2. In reply to comment 3, the author stated, “The protocol details plans for narrative synthesis grouped by outcome and exposure and potential meta analysis if the data is sufficiently homogeneous.” However, it remains unclear how the authors plan to summarize the evidence. Itemizing it as a “narrative synthesis” is not sufficient. What is the criterion for extracting evidence for narration? What is the evidence of interest? What do you define as evidence in the context of this study? These and others need clarification.

3. Upon reviewing lines 18-28 of the tracked file on Page 6, it is unclear whether the authors have clearly articulated the outcome of interest in this study, in keeping with PECO principles. The current write-up explains both diet and cognition variables as exposures. Please check again.

4. In response to comments 11& 12, and the referral on page 7, lines 7-33 in the tracked file, several unanswered questions remain. First, the outcome measures listed are screening tests and not outcomes. How do you expect to summarize evidence from the diverse screening test? How would cognition be defined? Poor? Good? Etc. What would be the basis? Defined by each study? Or defined by a predefined set of criteria> Third, I should add that these screening tests are viable instruments, but a physician determines the diagnosis to clarify the state of cognition. Please consider contextualizing these issues within this protocol.

5. My candid apologies for comment 16. That is not related to your protocol. Perhaps mixed writing with another review. Once again, apologies.

6. In reply to comment 18; the authors stated, “The full completed systematic review writeup will detail all confounding factors.” However, this comment highlights the need to clarify expected outcomes, i.e., juxtaposing the currently proposed review in light of the current trend in the literature, specifically regarding the approach, strength of evidence, and type of evidence likely to be produced by this review.

7. In reply to comment 19, the author replied that “As per normal practice, full details of limitations in this review will be provided in the review write-up.”, and referred the reviewer to page 2. It is obvious that the limitations of the review would be detailed in the completed review; however, the explicit comment offers the opportunity for the authors to identify potential challenges and issues likely to arise during the conduct of the review. Besides, itemizing the potential for no meta-analysis, there are other challenges not forecasted. For example, how would evidence be synthesized? It is not sufficient to mention that this is a narrative review. What would be the criteria for summarizing the evidence? This is not clear and could potentially be a limitation until all evidence is reviewed.

8. It is commendable that the authors provided an exhaustive introduction, search strategy, and reporting checklist, among other revisions carried out.

9. For future reference, the authors should clarify the line numbers where revisions were made in the response to reviewer comments file. Also, if possible, the section of the manuscript with the revisions may be copied and appended with the response to the reviewers' comments. By doing so, the reviewer follows your revision seamlessly.

**Reviewer**	**2**
**Name**	**Salama, Amany**
**Affiliation**	**Pharos University, Nutrition and food safty**
**Date**	**05-Nov-2025**
**COI**	

The authors have responded efficiently to all the editorial and reviewers' comments, amending all the previous flaws. Thank you.

VERSION 2 - AUTHOR RESPONSE

Dr. Thiel and Dr. Okekunle,

Thank you for the helpful and detailed feedback on our systematic review protocol. We have addressed all points raised and incorporated the necessary revisions into the manuscript,

providing specific justifications below. For clarification, the reviewer's comments are numbered. Our responses and actions are detailed directly below each comment.

1. "In reply to comment 1, the author states that 'The protocol specifies that this time limit was chosen to "enable the identification of recent high-quality studies that will be most beneficial for practice and policy decision makers" stated under the heading 'Electronic searches' Page 8.' However, it is highly speculative and subjective to conclude that only 'recent high-quality studies that will be most beneficial for practice and policy'. I suggest that the authors critically review this and revise the protocol accordingly."

Response: Thank you for the comment. The original protocol stated this time frame was chosen to "enable the identification of recent high-quality studies that will be most beneficial for practice and policy decision makers". However, we have now updated the comment to better reflect the reasoning behind this timeframe (Page 6, line 10-13).

Action: New sentence 'the identification of studies that reflect dietary intake in contemporary food environments. Hence, we focused on studies reported in the past 25 years.'

2. "In reply to comment 3, the author stated, 'The protocol details plans for narrative synthesis grouped by outcome and exposure and potential meta analysis if the data is sufficiently homogeneous.' However, it remains unclear how the authors plan to summarize the evidence. Itemizing it as a 'narrative synthesis' is not sufficient. What is the criterion for extracting evidence for narration? What is the evidence of interest? What do you define as evidence in the context of this study? These and others need clarification."

Response: Thank you for the comment. The protocol currently details plans to summarise the evidence in the 'Data synthesis' section where there is a detailed account of how the narrative synthesis will be grouped and sub-grouped. There is also clear clarification of the evidence of interest stated in the 'Data Synthesis' section (e.g., overall diet quality, specific macronutrient/food group intakes etc.) (page 8, lines 29&30).

Action: We have reworded that part of the 'Data synthesis' section to provide a clearer definition of our narrative synthesis plans (page 8, lines 24&25). We have also rearranged that section to detail the analysis method after the methods of data synthesis.

3. "Upon reviewing lines 18-28 of the tracked file on Page 6, it is unclear whether the authors have clearly articulated the outcome of interest in this study, in keeping with PECO principles. The current write-up explains both diet and cognition variables as exposures. Please check again."

Response: We thank the reviewer for identifying the typo when describing the PECO concept description.

Action: The protocol clearly states our PECO concepts under the ‘Electronic searches’ heading (page 5, lines 40 & 41).

4. "In response to comments 11& 12, and the referral on page 7, lines 7-33 in the tracked file, several unanswered questions remain. First, the outcome measures listed are screening tests and not outcomes. How do you expect to summarize evidence from the diverse screening test? How would cognition be defined? Poor? Good? Etc. What would be the basis? Defined by each study? Or defined by a predefined set of criteria? Third, I should add that these screening tests are viable instruments, but a physician determines the diagnosis to clarify the state of cognition. Please consider contextualizing these issues within this protocol."

Response: Thank you for your comment. We acknowledge and understand that some of the measures listed can be used as a screening test in clinical conditions/settings, however, they are also used as outcome measures in epidemiological studies therefore we are including them as outcome measures in this review. We would potentially include them if they were used in any academic epidemiological studies identified through our searches.

5. "My candid apologies for comment 16. That is not related to your protocol. Perhaps mixed writing with another review. Once again, apologies."
Response: Thank you. No further action required.

6. "In reply to comment 18; the authors stated, 'The full completed systematic review writeup will detail all confounding factors.' However, this comment highlights the need to clarify expected outcomes, i.e., juxtaposing the currently proposed review in light of the current trend in the literature, specifically regarding the approach, strength of evidence, and type of evidence likely to be produced by this review."

Response: Thank you for this comment. This systematic review protocol outlines the expected outcomes and results with its content.
Defining and specifying outcomes: Outcome measures of interest defined within the introduction of the protocol and specified within the ‘Types of outcome measures’ section with the methods. (page 6, lines 30-50 & page 7, lines 1-11).
Planned Data synthesis method: Data synthesis method and plan detailed in the section ‘Data synthesis’ (page 8, lines 9-37).
Risk of bias assessment: The methodological quality of all included studies will be systematically appraised using the relevant Joanna Briggs Institute (JBI) Critical Appraisal Checklists. This includes JBI Checklists for Randomized Controlled Trials, Quasi-Experimental

Studies, Cohort Studies, Case Control Studies, and Analytical Cross-Sectional Studies. To standardize the quality comparison across diverse study designs, each study will be allocated a final score of low or high risk of bias (page 8 lines 40-43 & page 9 lines 1-12). We feel adding a sentence/paragraph describing this again would add replication to the current content within the review.

7. "In reply to comment 19, the author replied that 'As per normal practice, full details of limitations in this review will be provided in the review write-up.', and referred the reviewer to page 2. It is obvious that the limitations of the review would be detailed in the completed review; however, the explicit comment offers the opportunity for the authors to identify potential challenges and issues likely to arise during the conduct of the review. Besides, itemizing the potential for no meta-analysis, there are other challenges not forecasted. For example, how would evidence be synthesized? It is not sufficient to mention that this is a narrative review. What would be the criteria for summarizing the evidence? This is not clear and could potentially be a limitation until all evidence is reviewed."

Response: Thank you for the comment. We acknowledge the need to explicitly identify potential challenges that may arise during the conduct of the review beyond the limitation that a meta-analysis is not expected due to anticipated heterogeneity. However, as this review is exploratory, it is usual practice to keep the approach to summary flexible until data extraction.

Action: As per reviewer question 2, we have updated the 'Data synthesis' section to provide a clearer definition of our narrative synthesis plans (page 8, lines 24&25).

8. "It is commendable that the authors provided an exhaustive introduction, search strategy, and reporting checklist, among other revisions carried out."

Response: Thank you. No further action required.

9. "For future reference, the authors should clarify the line numbers where revisions were made in the response to reviewer comments file. Also, if possible, the section of the manuscript with the revisions may be copied and appended with the response to the reviewers' comments. By doing so, the reviewer follows your revision seamlessly."

Response: Thank you for highlighting. We have adopted the practice of clarifying line numbers within this response to comments.

Thank you for your time and effort in reviewing this protocol.
